# Analyzing Statistical Mediation with Multiple Informants: A New Approach with an Application in Clinical Psychology

**DOI:** 10.3389/fpsyg.2015.01674

**Published:** 2015-11-13

**Authors:** Lesther A. Papa, Kaylee Litson, Ginger Lockhart, Laurie Chassin, Christian Geiser

**Affiliations:** ^1^Department of Psychology, Utah State University, LoganUT, USA; ^2^Department of Psychology, Arizona State University, PhoenixAZ, USA

**Keywords:** statistical mediation, indirect effects, multiple informants, multimethod design, multiple raters

## Abstract

Testing mediation models is critical for identifying potential variables that need to be targeted to effectively change one or more outcome variables. In addition, it is now common practice for clinicians to use multiple informant (MI) data in studies of statistical mediation. By coupling the use of MI data with statistical mediation analysis, clinical researchers can combine the benefits of both techniques. Integrating the information from MIs into a statistical mediation model creates various methodological and practical challenges. The authors review prior methodological approaches to MI mediation analysis in clinical research and propose a new latent variable approach that overcomes some limitations of prior approaches. An application of the new approach to mother, father, and child reports of impulsivity, frustration tolerance, and externalizing problems (*N* = 454) is presented. The results showed that frustration tolerance mediated the relationship between impulsivity and externalizing problems. The new approach allows for a more comprehensive and effective use of MI data when testing mediation models.

## Introduction

Clinical researchers are frequently interested in uncovering so-called *mediating* processes, in which an independent variable *X* produces effects on a mediator *M*, which subsequently influences an outcome variable *Y* (Baron and Kenny, 1986; MacKinnon, 2008; Hayes, 2009; Preacher and Kelley, 2011). Mediated effects are important to examine in clinical research because they help explain why and how treatments work (MacKinnon et al., 2013). For example, parent management training (*X*) has been shown to be effective for changing behavioral problems (*Y*) in children and adolescents by modifying parental behavior management practices (*M*; Weisz and Gray, 2008; Weisz and Kazdin, 2010). As another example, Arch et al. (2012) found both cognitive behavioral therapy and acceptance and commitment therapy (*X*) to positively influence patients’ anxiety levels (*Y*) through modifying anxiety sensitivity and cognitive defusion (*M*). The causal process underlying mediation models is naturally incorporated into both etiological and clinical theories (Chen, 1990; Kazdin and Nock, 2003; Kazdin, 2007, 2009, 2011).

**FIGURE 1:**
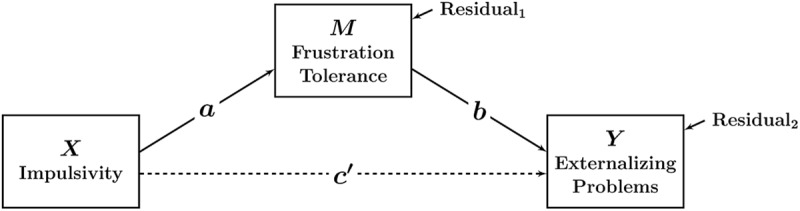
**Path diagram illustrating a mediation model with only observed variables.** In this model, statistical mediation is examined directly between observed variables (e.g., child reports). The parameters *a, b*, and *c*’ denote path (regression) coefficients. No latent variables or measurement error variables are included in the model.

Statistically, mediation is often analyzed through path analytic models. **Figure 1** illustrates the simplest mediation path model with one *X* variable, one mediator *M*, and one outcome variable *Y*. In this example, impulsivity (*X*) is hypothesized to indirectly affect externalizing problems through changing children’s frustration tolerance. In this model, higher levels of impulsivity are hypothesized to cause lower levels of frustration tolerance, which in turn elevate externalizing problems. The indirect (or mediated) effect can be quantified as *a*^∗^*b* (i.e., by taking the product of the two path coefficients *a* and *b*) and tested for statistical significance (e.g., MacKinnon, 2008)^1^.

In the present paper, we are concerned with statistical mediation analysis when multiple informant (MI) data is used to assess *X, M*, or *Y*. By MI data, we mean data obtained from different reporters or raters, such as, for example, children (i.e., self-reports), parents, teachers, or peers. The use of MI measurement designs is common and considered best methodological practice in clinical psychology (Achenbach et al., 1987; Rescorla et al., 2012; De Los Reyes et al., 2013). Using MI data allows researchers to obtain different perspectives and to determine the level of agreement (convergent validity) between different informants. Although we focus on different informants (raters, reporters) as different “methods” used to assess constructs or traits of interest in a mediation model, the statistical models presented in this article apply more generally to various “multi-method” measurement designs, in which methods could be reporters, tests, observations, physiological measures, etc. Therefore, we use the terms “informant” and “method” interchangeably in the present paper. We focus on informants in this paper given the wide-spread use of this “method” in clinical psychology (Achenbach et al., 1987; Rescorla et al., 2012; De Los Reyes et al., 2013, 2015).

In the case of MI measurement designs, clinical researchers face the challenge of how to meaningfully integrate MI data in statistical mediation models. In the present article, we review current approaches to MI mediation analyses in clinical research, discuss their advantages and limitations, present a novel latent variable approach to modeling MI mediation data, and show based on an application how this approach can improve the analysis of mediated effects in the context of MI data.

The issue of MI mediation analysis is important to investigate because (1) mediating pathways are among the most ubiquitous structural models in clinical research, (2) the use of MIs to assess a wide range of constructs is recommended and has become common practice, and (3) modern methods for testing mediated effects, including latent variable modeling techniques, continue to gain popularity in clinical research (MacKinnon et al., 2013). Due to the lack of clear guidelines about synthesizing these two methodological approaches (MI and mediation analysis), clinical researchers may be uncertain about how to best analyze mediation in the context of MI data. The goal of the present paper is to present a novel approach that combines recently developed confirmatory factor analysis (CFA) measurement models for MI data with modern mediation analysis to provide clinical researchers with better tools for studying mediation in the context of MI measurement designs.

## Current Approaches to Handling MI Data in Mediation Models

To provide an overview of current practices in integrating MI data in mediation models in clinical research, we conducted a Web of Science review of the top three journals by impact factor under the category ‘Psychology-Clinical.’ We included only journals that published original empirical mediation analyses using MI data (the three journals were: *Health Psychology, Journal of Abnormal Psychology*, and *Journal of Consulting and Clinical Psychology*). We restricted our review to the years 2004–2013 (i.e., the past 10 years since we began this study). Articles were selected for review if they tested at least one MI mediation model in which at least one variable in the model (i.e., *X, M*, or *Y*) was measured with at least two different types of informants. A total of 24 articles were obtained based on these criteria.

The most common approach identified in our review involved using composite scores based on averages of two or more informants’ reports and then performing mediation analyses on these newly computed composite variables (*N =* 22 studies or 91.7%)^2^. Six studies (25%) tested separate mediation models for each type of informant. Finally, four articles (16.7%) reported using different informants’ reports as separate indicators of latent variables in a latent variable [i.e., structural equation (SEM)] model.

### Models using Composite Scores of Averaged Reports

The composite score approach integrates MI data into a single statistical model and results in a single estimate of the mediated effect. An obvious advantage of this approach is its simplicity. On the other hand, this approach assumes that different raters’ reports should be weighted equally, which may not always be appropriate in practice. Most importantly, the composite score approach does not allow quantifying informant discrepancies (i.e., method effects) or examining the degree of convergent validity between informants.

### Models Separated by Informants

Reporting separate mediation analyses for each type of informant makes it unnecessary to combine potentially discrepant MI data. Rather than a single overall estimate of the mediated effect, a separate estimate is obtained for each type of informant. This approach can thus provide insights into whether data from different informants results in the same or different estimates for mediated effects. For example, mediated effects may be large and significant for one type of informant but not for another. In this situation, the researcher would have to decide which informant is most “trustworthy.”

One downside of this approach is that it does not integrate different informants’ reports into a single comprehensive statistical model, which may cause problems such as Type-I error inflation due to the use of multiple tests of significance (Kraemer et al., 2003). In addition, in this approach, the degree of between-informant discrepancy at the measurement level cannot be quantified or analyzed further.

### Latent Variable Structural Equation Models (SEMs)

**FIGURE 2:**
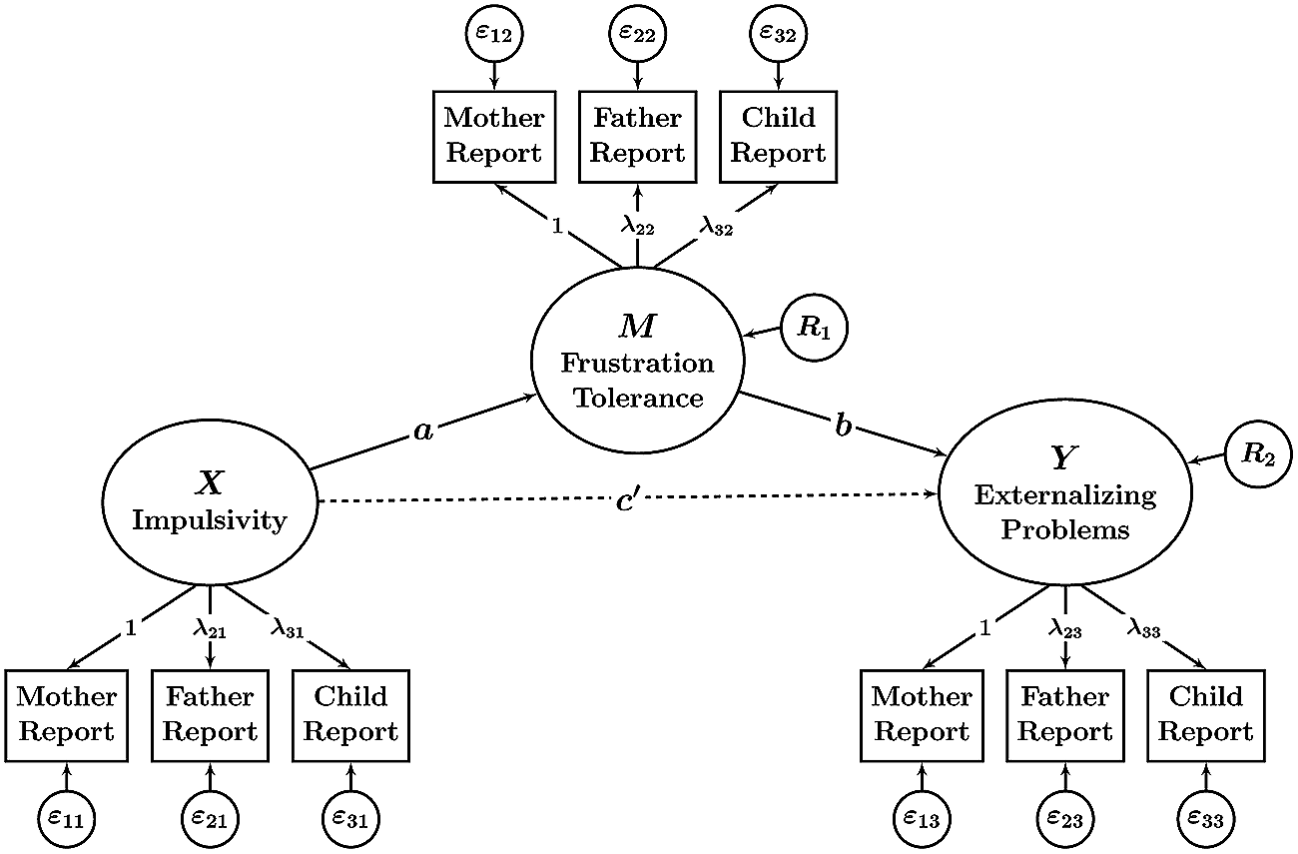
**Path diagram illustrating a mediation model with latent variables.** In this model, observed variables (different informants’ reports; shown in boxes) serve as indicators of latent variables (shown in ellipses). Statistical mediation is examined between latent variables that are corrected for measurement error and informant-specific effects. The parameters *a, b*, and *c*’ denote path (regression) coefficients. *R*_1_ and *R*_2_: latent residual variables. The parameters λ*_mt_* denote factor loadings (*m* = method or type of informant, *t* = trait). The model includes measurement error variables ε*_mt_* to account for random errors of measurement. The model does not include latent variables representing method (informant) effects (method factors).

The third most common approach to managing MI data in mediation models in our review was to use different informants’ observed scores as indicators of common latent variables as shown in **Figure 2**. An advantage of latent variable SEMs is that they separate true individual differences (true score variance in the sense of classical test theory) from variability that is caused by random measurement error (Bollen, 1989). Rather than modeling the mediated effect at the level of observed variables that are contaminated by measurement error (as done in the two previously discussed approaches), latent variable SEMs allow modeling mediated effects at the level of error-free latent variables (MacKinnon, 2008).

Furthermore, the latent variable SEM approach combines MI data into a single statistical model and allows each observed (informant-specific) variable to have a different factor loading on the common factor. The model is therefore able to model potential differences at the measurement level regarding how well each type of informant captures the content of the common latent variable. For example, in a given application, child reports may have smaller loadings than parent reports, indicating that children’s reports do not measure the common latent factor as well as do parent reports.

On the other hand, latent variable models that combine MI data by specifying a single common latent variable for *X, M*, and *Y*, respectively, do not allow separating informant-specific components of variance from random measurement error variance. This is because these models do not contain method factors for different informants. Instead, such models treat the systematic but informant-specific variance as part of the error variables. This can create bias in the measurement model by overestimating the amount of random error variance and underestimating an observed variable’s reliability (Eid, 2000). It also makes it impossible to study informant discrepancies in detail and to relate informant-specific variance components to external variables.

In summary, each of the three currently used approaches to MI mediation analysis in clinical psychology has advantages, but also several limitations that may impact the conclusions drawn from such analyses. Below we address some of these limitations by proposing a new latent variable modeling framework for MI mediation data that integrates modern CFA models for MI data with path analytic models for analyzing mediated effects.

## A Novel Approach to MI Mediation Analysis

Campbell and Fiske’s (1959) seminal paper on assessing convergent and discriminant validity with the so-called multitrait-multimethod (MTMM) matrix initiated a decades-long effort among methodologists to build measurement models that can (1) meaningfully integrate MI data into a single statistical model; (2) separate random error from systematic informant-specific effects by introducing latent method factors, (3) separate informant-specific (method) variance from variance that is shared across informants (convergent validity), and (4) be used to analyze trait-specific informant (method) effects (see Discussion below). Several types of CFA-MTMM models exist that have each of these qualities (for detailed discussions of different CFA-MTMM models, see Widaman, 1985; Marsh and Grayson, 1995; Wothke, 1995; Dumenci, 2000; Eid et al., 2006). Below we describe the extension of modern CFA-MTMM approaches to statistical mediation analysis in clinical research.

Eid (2000) developed a CFA-MTMM model that is known as the correlated traits-correlated (methods-minus-one) or CT-C(*M* – 1) model. In the present paper, we propose to extend the CT-C(*M* – 1) approach to an MI mediation model. We chose to use the CT-C(*M* – 1) approach for our extension to mediation analysis, because this approach has been shown to overcome a number of limitations of previous CFA-MTMM approaches. First, in contrast to most other approaches, the CT-C(*M* – 1) model uses latent variables that have been explicitly and clearly defined as conditional expectations or functions of conditional expectations of observed variables; as a result, all latent variables in the model have a clear meaning and interpretation (Eid, 2000; Eid et al., 2003; Geiser et al., 2008, 2014). Second, the CT-C(*M* – 1) model solves identification problems present in other models (Eid, 2000). Third, it has been shown that the CT-C(*M* – 1) model is well-suited for MI data obtained from structurally different (non-interchangeable) reporters (such as self-, parent, and teacher reports), which appear to be most common in psychology (Eid et al., 2008). We first provide a brief review of the standard CT-C(*M* – 1) model (without mediation) and then describe extensions to MI mediation models.

### The CT-C(*M* – 1) Approach

**FIGURE 3:**
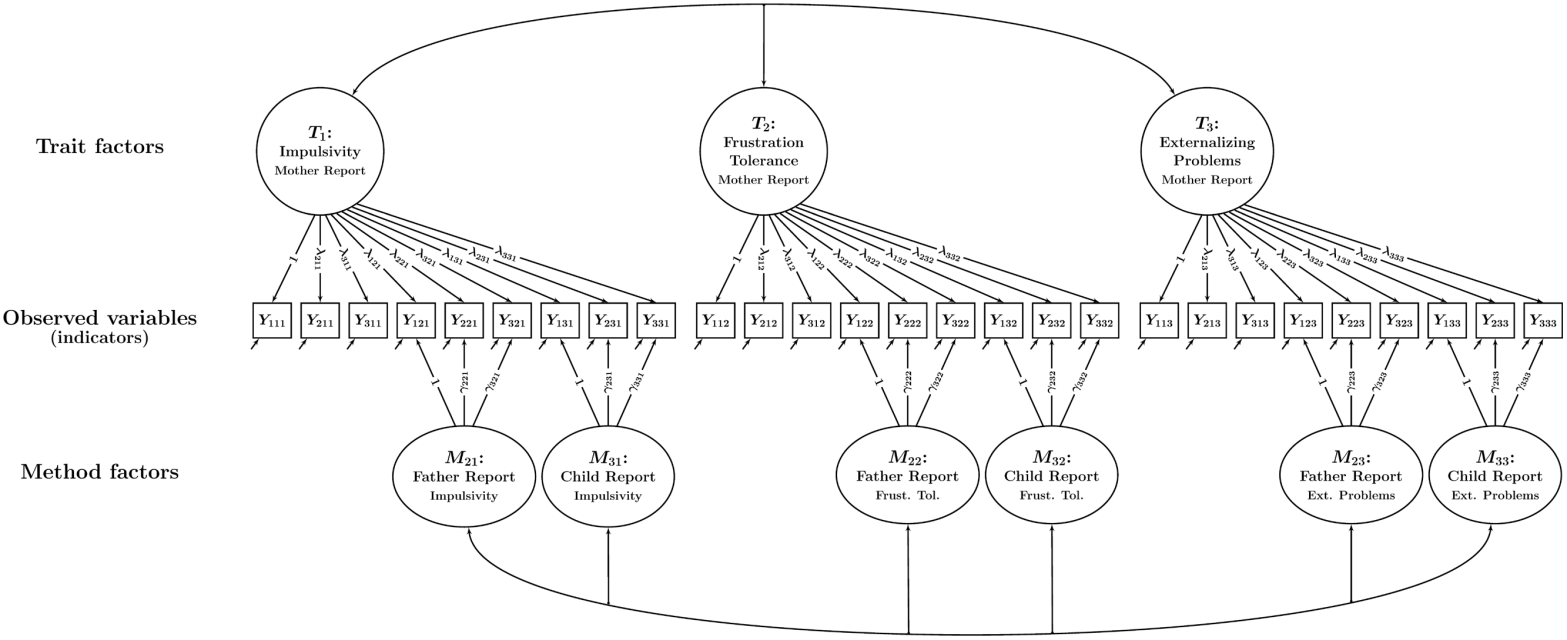
**Path diagram illustrating a CT-C(*M* – 1) model with a single trait factor per trait.** In the example, there are three traits and three methods. Each trait-informant combination is measured by three observed variables (indicators) *Y_imt_* (*i* = indicator, *m* = method or type of informant, *t* = trait). Trait factors *T_t_* are shown at the top, trait-specific method factors *M_mt_* at the bottom of the figure. The parameters λ*_imt_* and γ*_imt_* denote trait and method factor loadings, respectively. All trait factors can be correlated and all method factors can be correlated as indicated by double-headed arrows. Correlations between trait and method factors pertaining to the same trait are not allowed.

In the multiple-indicator CT-C(*M* – 1) approach (Eid et al., 2003), it is assumed that each type of informant (or method) provides information on each construct or “trait” (in the case of a mediation study, the traits would be *X, M*, and *Y*). Moreover, it is assumed that there are multiple observed variables (i.e., latent variable *indicators*; such indicators are often based on individuals’ responses to questionnaire or test items, [sub]scales, or item parcels) for each type of informant and trait. **Figure 3** shows a CT-C(*M* – 1) model for our example with three traits (impulsivity, frustration tolerance, and externalizing behavior) and three types of informants or “methods *m*” (mothers, fathers, and children). Each of the three types of informants provided ratings of the children’s behavior for each trait using three indicators (observed variables) per trait.

When the CT-C(*M* – 1) model is applied to MI data, one informant type is selected *a priori* to serve as a *reference informant*. For example, when dealing with mother, father, and child reports, mother reports might be selected as the reference informant (as shown in the example in **Figure 3**). We refer to the remaining informant types as non-reference informants (e.g., father- and self-reports in our example). These non-reference informants are contrasted against the reference informant. In this way, researchers can find out to which extent different informants’ reports converge with a reference or “gold standard” informant (in our example: father- and self-reports with mother reports).

There are different approaches to choosing an appropriate reference informant. Often, a reference informant is chosen based on theory (e.g., a theory may predict that a specific informant type is particularly trustworthy or valid; see De Los Reyes and Kazdin, 2005 for guiding principles of informant selection), a researcher’s prior experience with different informants, informants’ access to the specific construct being assessed (e.g., self-reports may be more valid than other-reports for assessing more covert constructs such as depression, whereas other-reports may be more valid for assessing certain types of overt behaviors such as aggression), the most typical or established informant used in a given field (e.g., mother reports for child behavior problems; Bechtold et al., 2013) or other assessment standards.

It can be seen that the model in **Figure 3** contains a latent variable for each trait. These latent variables are referred to as *trait factors*. All (reference and non-reference) indicators load onto the trait factors. Furthermore, the non-reference indicators load onto additional latent variables that are specific to each informant and trait. These additional latent variables are referred to as method factors. As can be seen from **Figure 3**, there are separate father and child method factors for impulsivity, frustration tolerance, and externalizing problems, respectively. There are no method factors for the reference indicators. Therefore, the trait factors represent the common factors or “true score variables” pertaining to the reference informant (mother reports; Eid et al., 2003). The method factors are defined as residual factors with regard to the trait factors that pertain to the same construct. The method factors are therefore by definition uncorrelated with the trait factors pertaining to the same trait and capture systematic residual variance in the non-reference indicators that is not shared with the reference indicators. For example, the method factor for father reports of impulsivity represents that portion of father reports that is neither shared with mother reports of impulsivity nor due to measurement error.

Given that trait factors, method factors, and error variables represent independent sources of observed informant variance in the CT-C(*M* – 1) model, we can decompose the observed score variance V ar(Y _imt_) and true score variance V ar(τ_imt_) of each variable as follows:

$$\begin{array}{c} Var(Y_{imt})=Var(\tau_{imt})+Var(\varepsilon_{imt})\;\\ \begin{array}{c} =\;\lambda_{imt}^{2}Var(T_{t})+\;\gamma_{imt}^{2}Var(M_{mt})+Var(\varepsilon_{imt})\\ Var(\tau_{imt})=Var(Y_{imt})-Var(\varepsilon_{imt})\;\\ =\;\lambda_{imt}^{2}Var(T_{t})+\;\gamma_{imt}^{2}Var(M_{mt}).\; \end{array} \end{array}$$

Here, the index *i* indicates the *i*th observed variable (indicator), *m* indicates the *m*th method (i.e., informant type), and *t* indicates the *t*th trait or construct that is being measured. The variable *τ_imt_* is the true score variable that represents that portion of an observed variable *Y_imt_* that is free of measurement error. *T_t_* and *M_mt_* refer to the trait and method factors, respectively, and *ε_imt_* denotes a measurement error variable. Trait and method factor loadings are indicated by λ*_imt_* and γ*_imt_*, respectively.

To quantify the proportion of true score variance in a given variable that is shared with the reference method, the consistency coefficient *CO* can be calculated from estimated model parameters:

$$CO=\frac{\lambda_{imt}^{2}Var(T_{t})}{\lambda_{imt}^{2}Var(T_{t})+\;\gamma_{imt}^{2}Var(M_{mt})}=\frac{\lambda_{imt}^{2}Var(T_{t})}{Var(Y_{imt})-Var(\varepsilon_{imt})}.$$

The method-specificity coefficient *MS* gives the proportion of true score variance that is unique to a given rater type (not shared with the reference method):

$$MS=\frac{\gamma_{imt}^{2}Var(M_{mt})}{\lambda_{imt}^{2}Var(T_{t})+\gamma_{imt}^{2}Var(M_{mt})}=\frac{\gamma_{imt}^{2}Var(M_{mt})}{Var(Y_{imt})-Var(\varepsilon_{imt})}.$$

Note that *CO* and *MS* add up to 1 (100% true score variance). In contrast to *CO* and *MS*, the reliability coefficient *Rel* indicates the proportion of *observed* variance that is due to systematic sources of variance (trait or method factors) and not due to measurement error:

$$\begin{array}{c} Rel=\frac{\lambda_{imt}^{2}Var(T_{t})+\gamma_{imt}^{2}Var(M_{mt})}{\lambda_{imt}^{2}Var(T_{t})+\gamma_{imt}^{2}Var(M_{mt})+Var(\varepsilon_{imt})}\\ =\frac{\lambda_{imt}^{2}Var(T_{t})+\gamma_{imt}^{2}Var(M_{mt})}{Var(Y_{imt})}.\; \end{array}$$

The CT-C(*M* – 1) model has several strengths for the analysis of MI data. By including method factors in addition to trait factors, the model allows researchers to properly separate true convergent validity from true method specificity and random measurement error. Including method factors has the additional benefit that informant effects are captured by latent variables. Method factors can be related to external variables to explain informant discrepancies (e.g., gender and age). By using multiple indicators within each trait-informant combination, the model enables researchers to specify trait-specific method factors. *Trait-specific* means that method effects for the same type of reporter (e.g., underestimation of impulsivity relative to the reference informant) do not have to be perfectly correlated across different constructs. This is beneficial when informant effects differ for different traits, which is typically the case in practice (Marsh and Hocevar, 1988; Eid et al., 2003). For example, father’s underestimation of impulsivity relative to mother reports is not necessarily perfectly correlated with father’s underestimation of externalizing problems relative to mother reports. Additional advantages of the CT-C(*M* – 1) model in relation to other CFA-MTMM models are discussed in Geiser et al. (2008) as well as Geiser et al. (2014).

### The CT-C(M – 1) Approach with Indicator-specific Traits

The use of multiple indicators within each trait-informant combination is useful because it enables researchers to allow for and examine trait-specific method effects. Single-indicator models do not allow for trait-specific method effects and thus make the often unrealistic assumption that method effects generalize perfectly across traits for all methods. This can lead to bias in the modeling results.

When multiple indicators are used, these indicators may not be perfectly unidimensional in the sense of classical test theory (i.e., they might contain item- or scale-specific variance that may generalize across different informants). Even seemingly minor differences in item wording or content can cause such variable-specific effects. The CT-C(*M* – 1) model with a single trait factor per trait (as shown in **Figure 3**) implicitly assumes that there are no variable-specific effects and may thus not fit well when indicators are rather heterogeneous.

**FIGURE 4:**
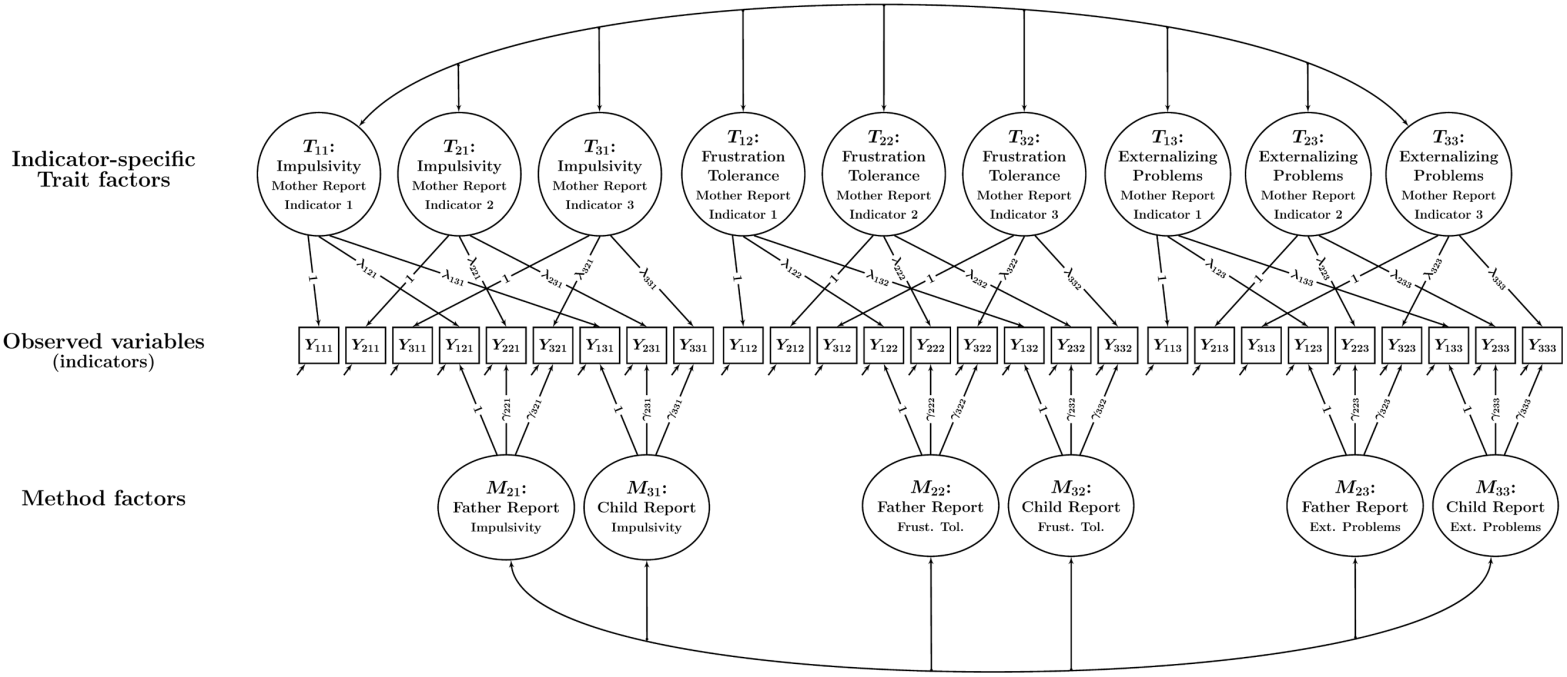
**Path diagram illustrating a CT-C(*M* – 1) model with indicator-specific trait factors.** In the example, there are three traits and three methods. Each trait-informant combination is measured by three observed variables (indicators) *Y_imt_* (*i* = indicator, *m* = method or type of informant, *t* = trait). Trait factors *T_it_* are shown at the top, trait-specific method factors *M_mt_* at the bottom of the figure. Observed variables with the same index *i* and *t* (but different method index *m*) load onto the same indicator-specific trait factor *T_it_*. The parameters λ*_imt_* and γ*_imt_* denote trait and method factor loadings, respectively. All trait factors can be correlated and all method factors can be correlated as indicated by double-headed arrows. Correlations between trait and method factors pertaining to the same trait are not allowed.

This problem can be solved by using a CT-C(*M* – 1) model version with indicator-specific trait factors that accounts for variable-specific effects (Eid et al., 2008). This model version is shown in **Figure 4**. It can be seen that in the model with variable-specific trait factors, each observed variable has its own trait factor. The variable-specific trait factors can be correlated, but need not be *perfectly* (1.0) correlated—as is implicitly assumed in the single-trait model version. The variable-specific trait model is therefore less restrictive and preferred in situations in which researchers work with items or scales that are not perfectly unidimensional. High correlations between the variable-specific traits within the same construct indicate a high degree of homogeneity of the indicators, whereas low correlations indicate that the indicators reflect rather different aspects or facets of the construct.

## Ct-C(*M* – 1) MI Mediation Model

**FIGURE 5:**
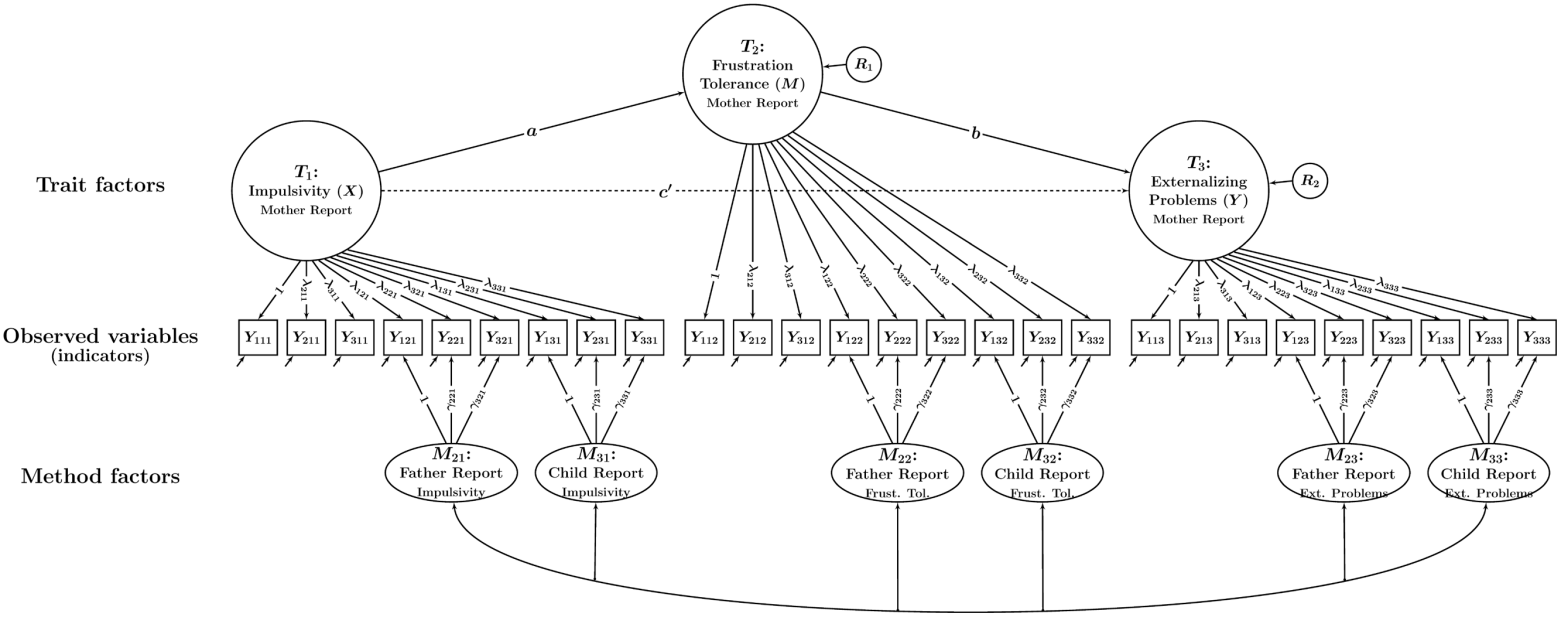
**Path diagram illustrating a CT-C(*M* – 1) mediation model with a single trait factor per trait.** In this model, statistical mediation is examined between latent trait factors. The parameters *a, b*, and *c*’ denote path (regression) coefficients. *R*_1_ and *R*_2_: latent residual variables. All other parameters are the same as in **Figure 3**.

In the present paper, we propose to combine CT-C(*M* – 1) measurement models with structural mediation models used in path analysis and conventional (single-informant) SEMs. **Figure 5** illustrates a combined CT-C(*M* – 1) mediation model with global traits that can be used for homogenous indicators. It can be seen that in contrast to the conventional CT-C(*M* – 1) model, the CT-C(*M* – 1) mediation model involves structural regression paths between the latent trait factors that represent *X, M*, and *Y* in line with conventional mediation analysis.

**FIGURE 6:**
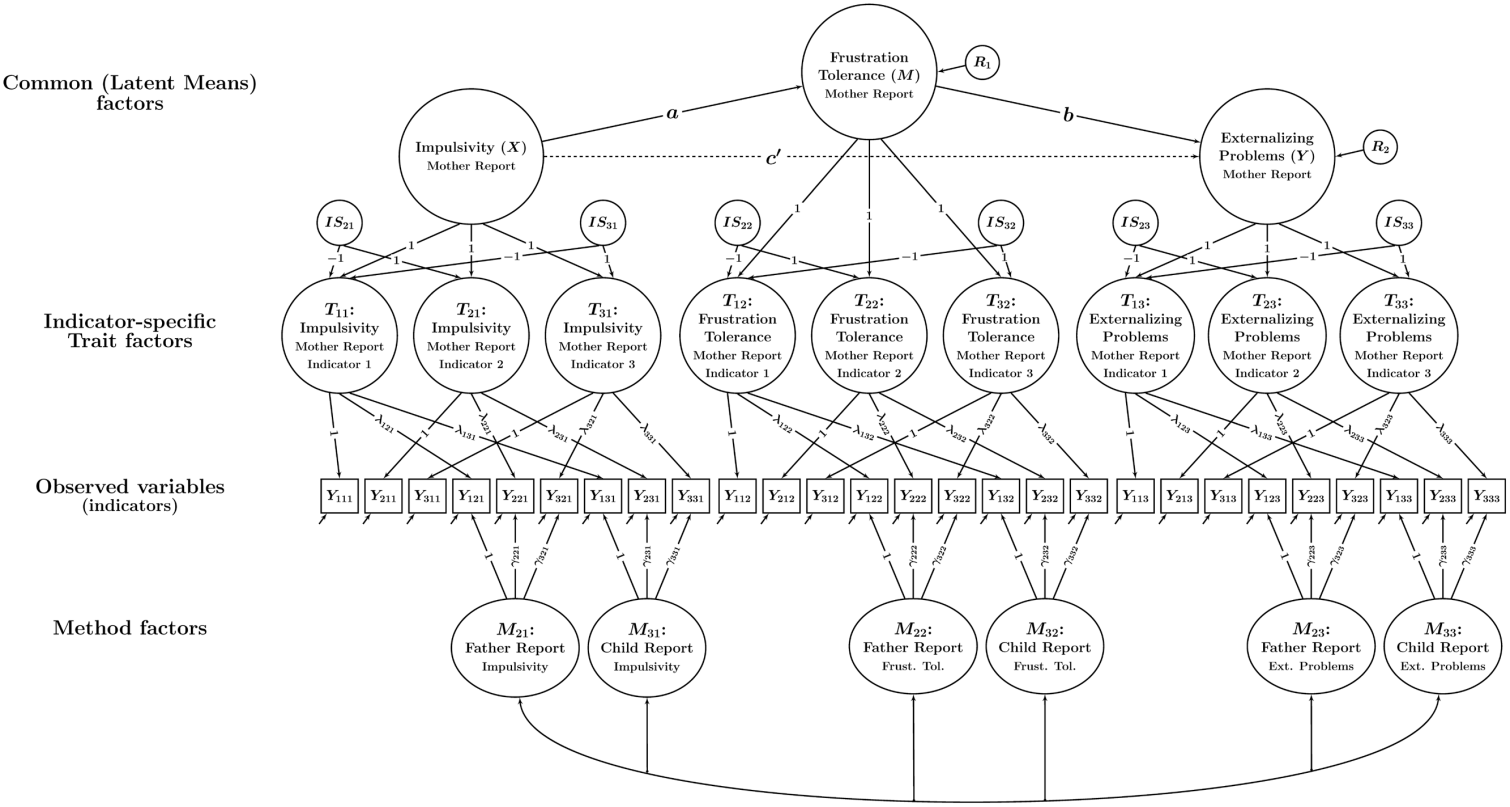
**Path diagram illustrating a CT-C(*M* – 1) mediation model with indicator-specific trait factors and a latent means approach used to aggregate indicator-specific trait factors.** In this model, statistical mediation is examined between common trait (latent means) factors that represent averages of indicator-specific trait factors pertaining to the same trait. The latent variables *IS_it_* represent deviations of the indicator-specific trait factors from the average and reflect parcel-specific effects. All other parameters are the same as in **Figure 4**.

**Figure 6** shows the variable-specific trait factor version of the CT-C(*M* – 1) mediation model. Given that the indicator-specific model version contains as many trait factors as there are indicators, the question arises as to how mediated effects should be analyzed in this version of the model. One possibility is to specify separate mediation models for some or all indicator-specific trait factors (this option is *not* shown in the figure). This would make sense in cases in which the indicators are (either theoretically or empirically) highly distinct in terms of their content or the facets of the constructs that they are reflecting. In this case, there might be theoretically anticipated differences in the mediated effects between indicators, possibly warranting separate mediation analyses for different indicator-specific trait factors.

One obvious downside of analyzing mediated effects separately for each indicator-specific trait factor is that this leads to a large number of possible combinations of *a* and *b* paths. When all possible combinations of *a* and *b* paths are analyzed, a researcher would end up estimating and testing a total of 27 mediated effects. This is clearly not practical—and also typically not necessary. We recommend this approach only when a researcher has clear *a priori* theoretical hypotheses regarding differences in the mediated effects across indicators.

### Aggregating Variable-specific Traits: The Latent Means Approach

In many practical applications, indicators are designed to be (essentially) homogenous and differ only in minor ways regarding their content. In these cases, it will be more practical for researchers to analyze only a single mediation model (while still properly accounting for statistical differences between indicators). We now present a way to combine the variable-specific trait factors that is appropriate when indicators are essentially homogenous. For this purpose, we use a so-called latent means modeling approach in which common factors are defined as averages of the variable-specific trait factors (Pohl and Steyer, 2010; Geiser et al., 2014). The basic idea of the latent means approach is to define or “construct” a common factor as the average of variable-specific trait factors. In this way, a researcher can reduce the number of trait factors to be analyzed in the mediation model, while still properly accounting for variable-specific effects (i.e., differences between indicators). The equation below illustrates how three variable-specific trait factors can be aggregated to a single common trait factor for a given trait *t* (where *t* indicates *X, M*, or *Y*):

*Common factor_t_* = (*Trait*_1_*_t_* + *Trait*_2_*_t_* + *Trait*_3_*_t_*)/3.

In **Figure 6**, this is shown for each of the three constructs, resulting in three common (latent means) factors representing *X, M*, and *Y* in the mediation model. Each of the three variable-specific trait factors can deviate from the average, reflecting variable-specific effects. These variable-specific effects are captured in so-called indicator-specific factors *IS_it_* that are defined as the deviations of a variable-specific trait factor from the common factor:

*IS*_1_*_t_* = *Trait*_1_*_t_* – *Common factor_t_*

*IS*_2_*_t_* = *Trait*_2_*_t_* – *Common factor_t_*

*IS*_3_*_t_* = *Trait*_3_*_t_* – *Common factor_t_*.

By definition, the sum of all deviations from the average equals zero, hence *IS*_1_*_t_* + *IS*_2_*_t_* + *IS*_3_*_t_* = 0. It follows that, for example, *IS*_1_*_t_* = (–*IS*_2_*_t_* –*IS*_3_*_t_*). Given that each *IS* factor can be written as a deterministic function of the two remaining *IS* factors, it is sufficient to include only two *IS* factors per trait in the model. Here, without loss of generality, we chose to drop *IS*_1_*_t_*, so that we obtain:

*Trait*_1_*_t_* = *Common factor_t_* – *IS*_2_*_t_* – *IS*_3_*_t_*

*Trait*_2_*_t_* = *Common factor_t_* + *IS*_2_*_t_*

*Trait*_3_*_t_* = *Common factor_t_* + *IS*_3_*_t_*.

This specification is depicted in **Figure 6** with the signs of the loadings of the *IS* factors reflecting the implicit weights of +1 or –1 in the above equations. **Figure 6** shows that by aggregating the three variable-specific traits for each construct, we can analyze a single mediated effect based on the common factors, which simplifies the modeling considerably.

Both versions of the CT-C(*M* – 1) mediation model can be applied to either cross-sectional or longitudinal mediation studies. Below we present an empirical illustration of the new approach.

## Method

### Sample

The data for this example are from a larger multirater study of the intergenerational effects of alcohol disorder (see Chassin et al., 1991, for details). The study was approved by Arizona State University’s internal review board. For simplicity, and given that the present application is for illustrative purposes rather than drawing substantive conclusions, we used cross-sectional data in the example presented here. We encourage researchers to use longitudinal designs in the study of mediation in line with what others have recommended (Cole and Maxwell, 2003; Preacher, 2015). The sample consisted of *N* = 454 children (mean age = 12.7; 47.1% female), for which mother, father, and self-reports were collected. In line with our running example, we examined a mediation model with three constructs: impulsivity (*X*), frustration tolerance (*M*), and externalizing problems (*Y*). In an actual empirical study, the findings could be used, for example, to determine whether targeting youths’ frustration tolerance in a clinical setting is a viable approach to reducing levels of youths’ psychopathology (Roosa et al., 1997).

### Measures

Children, mothers, and fathers responded to face-to-face structured interview items for each construct. *Impulsivity* was measured with a 12-items subscale of the Emotionality, Activity, Sociability, and Impulsivity Scale (Buss and Plomin, 1984). An example item within this subscale includes ‘I/[Target Child] generally seek[s] new and exciting experiences and sensations’; respondents indicated the extent to which each statement was like the target child on a scale from 1 (Very Unlike Me/Him/Her) to 5 (Very Like Me/Him/Her). *Frustration Tolerance* was measured with an adapted version of the nine-items Frustration Tolerance subscale of the Teacher Child Rating Scale (Hightower et al., 1989). An example item includes ‘I/[Target Child] accept[s] things that don’t go my/[his/her] way’; responses were modified to be consistent with the five-point scale of the Impulsivity subscale as described above. *Externalizing Problems* within the past 3 months were measured with the Externalizing dimension of the widely used Youth Self Report and Child Behavior Checklist (YSR and CBCL; Achenbach and Ruﬄe, 2000). Children reported on 21 items within this dimension of the YSR, while mothers and fathers reported on 31 items within the CBCL. Details of sample recruitment and representativeness are reported elsewhere (Chassin et al., 1991, 1992).

### Statistical Modeling

As explained above, the CT-C(*M* – 1) approach requires multiple (at least two) observed variables for each trait-informant combination to include trait-specific method factors. To obtain multiple indicators, we created three composite scores (i.e., item parcels) for each scale within each of the three informants (rather than creating a single composite; a general discussion of the merits and limitations of item parceling can be found in Little et al., 2002)^3^. For the creation of the parcels, we selected items that were equivalent across informants. We used three indicators per trait-informant combination because this ensures the identification of all latent factors even in cases in which factors happen to not be correlated with other factors.

All analyses were carried out in Mplus 7 (Muthén and Muthén, 1998–2012) using maximum likelihood estimation. A sample Mplus input file for the final model can be found in Appendix A. Preliminary analyses revealed that there were slight parcel-specific effects, allowing us to illustrate the more complex indicator-specific trait version of the CT-C(*M* – 1) model that accounts for variable-specific effects. Given that parcel-specific effects were relatively weak in the present study (meaning the parcels were essentially homogeneous), we did not expect systematic differences between the item parcels in terms of the mediated effect. We therefore applied the latent means approach described above to aggregate the indicator-specific trait factors into composite latent mean factors in line with **Figure 6**. As a result, only a single latent mediation model had to be tested. In order to test the mediated effect for statistical significance, we computed 95% confidence intervals based on the bias-corrected bootstrap method with 1000 draws to generate confidence intervals as has been recommended for conventional mediation analyses (MacKinnon et al., 2004; also see Hayes and Scharkow, 2013, for detailed comparisons of resampling methods for testing indirect effects).

## Results

**FIGURE 7:**
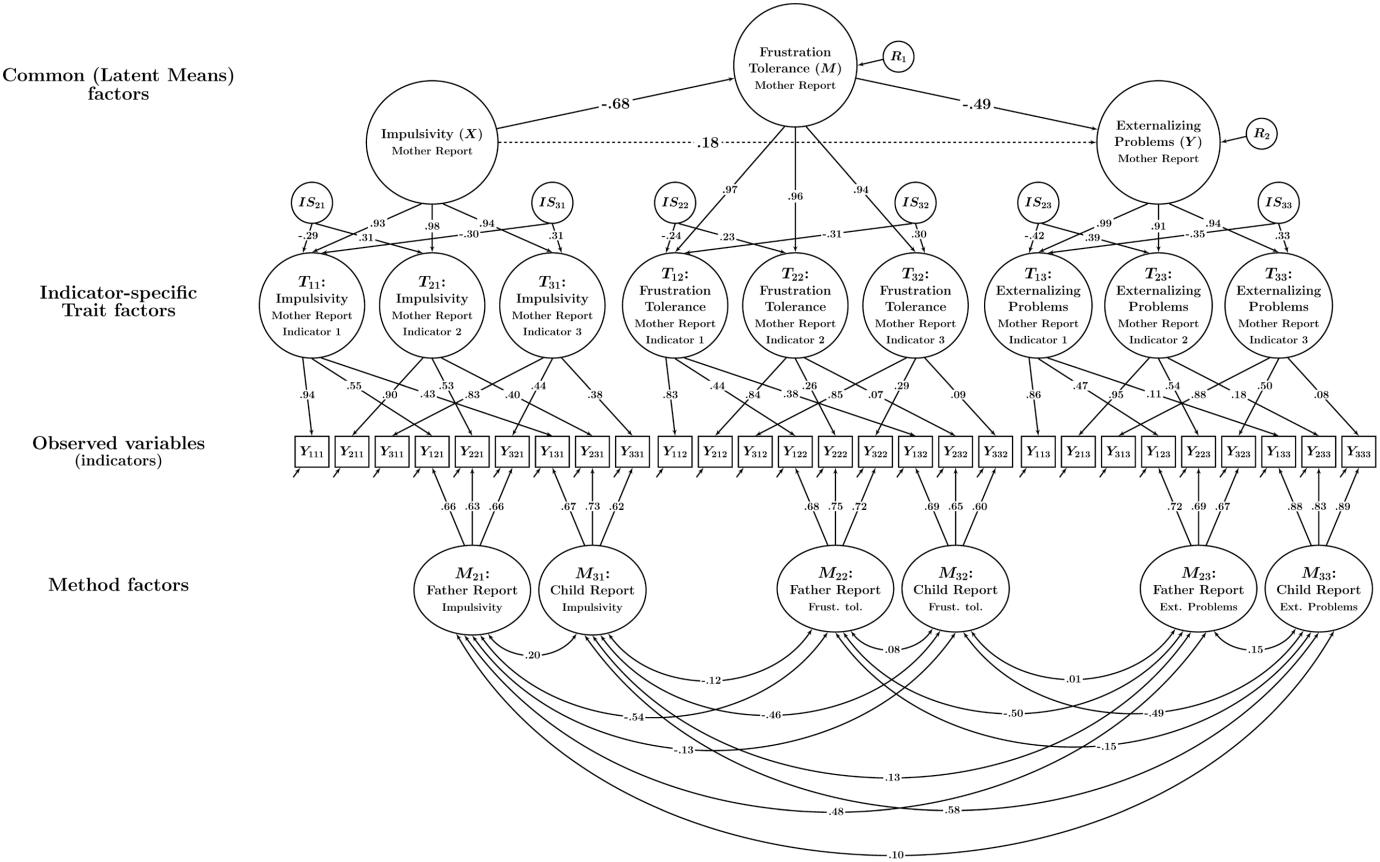
**Path diagram illustrating the CT-C(*M* – 1) mediation model in **Figure 6** with standardized parameter estimates obtained for the present data**.

The multiple-indicator CT-C(*M* – 1) mediation model showed a good fit to the data, χ^2^(219, *N* = 447) = 292.32, *p* < 0.001, RMSEA = 0.03, CFI = 0.99, SRMR = 0.04. The consistency, method-specificity, and reliability coefficients (averaged across item parcels) are presented in **Table 1**. Standardized parameter estimates are shown in **Figure 7**. From the consistency coefficients, it can be seen that mother and father rating shared a substantial portion of true score variance for the constructs impulsivity (on average 38% shared true score variance) and externalizing problems (on average 34% shared true score variance), indicating relatively high levels of convergent validity. In contrast, mother and father ratings shared only about 13% of true score variance for frustration tolerance, indicating low levels of convergent validity for this construct. Child ratings only showed moderate convergence with mother reports for impulsivity (on average 27% shared true score variance) and virtually no convergence with mother reports for frustration tolerance (4%) and externalizing problems (2%). Reliability estimates ranged from very low values (e.g., for child reports of frustration tolerance) to rather high values for externalizing behavior and mother reports of impulsivity. The rather mixed and partly low reliability estimates in this example underline the importance of using latent variable models that allow estimating mediated effects at the level of latent variables that are corrected for measurement error.

**Table 1:** Consistency, method-specificity, and reliability estimates obtained from the CT-C(*M* – 1) mediation model.

**Informant**	**Consistency**	**Method-specificity**	**Reliability**
	
	**Impulsivity**		
Mother			0.79 (0.67, 0.89)
Father	0.38 (0.30, 0.42)	0.62 (0.58, 0.70)	0.69 (0.63, 0.74)
Child	0.27 (0.25, 0.31)	0.73 (0.69, 0.75)	0.61 (0.52, 0.69)
	**Frustration tolerance**		
Mother			0.75 (0.71, 0.80)
Father	0.13 (0.12, 0.13)	0.87 (0.87, 0.88)	0.65 (0.61, 0.70)
Child	0.04 (0.01, 0.10)	0.96 (0.90, 0.99)	0.44 (0.41, 0.48)
	**Externalizing behavior**		
Mother			0.80 (0.75, 0.88)
Father	0.34 (0.33, 0.35)	0.66 (0.65, 0.67)	0.74 (0.70, 0.77)
Child	0.02 (0.01, 0.04)	0.98 (0.96, 0.99)	0.79 (0.76, 0.81)

*Entries represent average values across parcels. Values in parentheses give the ranges. Consistency and method-specificity coefficients are given for the true score variables and thus add up to 1 within each trait-informant combination. Mother reports served as reference method in this model. Therefore, consistency and method-specificity estimates for father and child reports are interpreted relative to mother reports.*

Correlations between the trait-specific method factors within the same type of informant (father and child reports) indicated only a moderate degree of generalization of informant effects across constructs for both father and child reports. The absolute values of the correlations ranged between *r* = 0.48 and 0.54 for father reports and between *r* = 0.46 and 0.58 for child reports, and were thus far from 1.0. This shows that in the present application, informant biases relative to mother reports were largely construct-specific.

Correlations between father and child method factors within the same constructs were small (*r* = 0.20 for impulsivity, *r* = 0.08 for frustration tolerance, and *r* = 0.15 for externalizing problems). This showed that fathers’ and children’s unique perspectives (i.e., their deviation from mothers’ perspective) were only to a small extent shared across these two rater types. In other words, fathers’ and children’s deviations from mothers’ view represented mostly a unique father or child informant perspective rather than a common deviation from mothers’ views.

The results of the mediation analysis revealed a small direct path from the impulsivity factor to externalizing behavior factor. The *c*′ path was barely significant at the 0.05 level in the unstandardized solution (unstandardized *c*′ estimate = 0.08 [95% CI: 0.004, 0.14]) and non-significant in the standardized solution (standardized *c*′ estimate = 0.18, [95% CI: -0.05, 0.40]). The indirect path from impulsivity to externalizing problems via frustration tolerance was significant at the 0.05 level (unstandardized *a*^∗^*b* estimate = 0.14 [95% CI: 0.10, 0.21], standardized *a*^∗^*b* estimate = 0.34 [95% CI: 0.13, 0.55]). The indirect path accounted for about 65% of the total effect (sum of direct and indirect paths) from impulsivity to externalizing problems. About 47% of the variance in the frustration tolerance factor was accounted for by the impulsivity factor. About 40% of the variance in the externalizing problems factor was accounted for by the impulsivity and frustration tolerance factors combined.

## Discussion

Statistical mediation analyses are used to identify mechanisms of change and are therefore of great interest to clinical researchers. In addition, collecting MI data is considered methodological best practice in clinical psychology (Hunsley and Mash, 2007). When MI data is used in studies of statistical mediation, the question arises as to how the data can be most properly analyzed. We reviewed methods that are currently used for this purpose in clinical psychology and described some of their advantages and limitations. We then presented a new latent variable approach to studying mediation with MIs that overcomes some of the limitations of currently used approaches. In our discussion, we describe the advantages and limitations of our approach and outline potential future extensions.

### Advantages

The CT-C(*M* – 1) mediation model integrates the information from MIs and multiple indicators into a comprehensive statistical model. The CT-C(*M* – 1) approach uses latent variables and thus allows researchers to correct for random error in the measurements. In contrast to MI mediation models previously used in clinical psychology, the CT-C(*M* – 1) mediation model uses trait and method factors to explicitly separate trait effects, informant effects, and measurement error. It therefore allows clinical researchers to quantify the convergent validity (consistency), method (informant) specificity, and reliability of different informants’ reports. By using multiple indicators for each informant type and trait, method factors can be specified as trait-specific. Therefore, the model can be used to study to which extent informant discrepancies (1) generalize across different traits (e.g., impulsivity vs. frustration tolerance) and (2) are shared between different informants (e.g., fathers and children) for a given trait. Another benefit of representing informant discrepancies in terms of latent method factors is that these effects could be related to other variables to study such discrepancies in greater detail (i.e., method factors could be correlated with external variables such as gender, age, etc.). Furthermore, the model version with variable-specific trait factors permits for the possibility that different indicators of a trait may not be perfectly homogenous. In this way, potential additional method effects that may arise due to differences in item wording or content differences between scales can be properly modeled. Mediated effects can be studied at the latent level, either among variable-specific traits or among general trait factors.

Finally, although the present paper focused on an example in which *X, M*, and *Y* were all assessed with MI (and with the same types of informants), the models presented here are flexible and can also be applied in situations in which only some constructs are assessed with MI or in situations in which different types of informants provide ratings for different constructs. For example, the present models can also be applied in situations in which the *X* variable is a treatment condition (and hence not measured by MI; MacKinnon et al., 2013) and only *M* or *Y* are measured by MI.

### Limitations

The CT-C(*M* – 1) mediation model is more complex than previously used methods. It requires the use of multiple indicators for each trait-informant combination and uses complex latent variable statistical methodology. Using multiple indicators can create additional complexities, as shown in our illustrative application. In the case of heterogeneous indicators, a researcher either has to deal with many variable-specific trait factors or with a somewhat non-standard approach to combining indicator-specific trait factors into three common trait factors.

Another specialty of the CT-C(*M* – 1) mediation model is that it uses a reference informant approach to integrating MI data into a comprehensive statistical model. This reference approach is similar to dummy coding in regression analyses with a reference category. Even though the choice of the reference informant is often straightforward, researchers may not always find it easy to make a case for a particular type of informant to serve as reference. In our illustrative example, we selected mother reports as reference, because mothers often spend the most time with the children and because mothers’ reports are often the primary source of information in clinical studies.

Other researchers have argued that there is often no gold standard measure in clinical settings, thus rendering an “optimal-informant” approach difficult (Kraemer et al., 2003). As an alternative, Kraemer et al. (2003) proposed the use of selected informants representing specific perspectives and contexts as well as principal component analysis based aggregation techniques to extract trait, context, and informant-specific components of variance. Applying their approach to statistical mediation analysis could be an interesting alternative in cases in which an optimal-informant approach is not feasible.

## Conclusion

The present models can help researchers overcome several limitations of previous MI mediation models. We hope that clinical researchers will find the new approaches useful to further improve clinical research and practice.

## Conflict of Interest Statement

The authors declare that the research was conducted in the absence of any commercial or financial relationships that could be construed as a potential conflict of interest.

## Footnotes

1 Though other commonly used mediation tests such as the causal steps approach first proposed by Baron and Kenny (1986) do not estimate a single mediation parameter, we focus on the *a^∗^b* mediated effect and corresponding confidence intervals because of simulation work showing their enhanced power (Fritz and MacKinnon, 2007) and reduced bias (MacKinnon et al., 2004).

2 Note that the percentages of types of MI mediation analyses do not add to 100% because some articles used more than one technique.

3 To create parcels, exploratory factor analyses were conducted at the item level to determine the extent to which items loaded on a single factor for each of the three constructs of interest: impulsivity, frustration tolerance, and externalizing behavior problems. Analyses were conducted separately for mother, father, and child ratings for each construct of interest. Findings from the mother analyses were used to create the item parcels. Items were distributed across parcels based on the magnitude of their factor loadings such that the item with the highest factor loading would be paired with the item with the lowest factor loading and the next highest would be paired with the next lowest and so on. Each pair was assigned to a parcel until all items were utilized. Within each construct, the parcels contained the same number of items.

## References

[B1] Achenbach T. M. Achenbach T. M. McConaughy S. H. Howell C. T. (1987). Child/adolescent behavioral and emotional problems: implications of cross-informant correlations for situational specificity. *Psychol. Bull.* 101 213–232. 10.1037/0033-2909.101.2.213 3562706

[B2] Achenbach T. M. Ruﬄe T. M. (2000). The Child Behavior Checklist and related forms for assessing behavioral/emotional problems and competencies. *Pediatr. Rev.* 21 265–271. 10.1542/pir.21-8-265 10922023

[B3] Arch J. J. Wolitzky-Taylor K. B. Eifert G. H. Craske M. G. (2012). Longitudinal treatment mediation of traditional cognitive behavioral therapy and acceptance and commitment therapy for anxiety disorders. *Behav. Res. Ther.* 50 469–478. 10.1016/j.brat.2012.04.007 22659156

[B4] Baron R. M. Kenny D. A. (1986). The moderator-mediator variable distinction in social psychological research: conceptual, strategic, and statistical considerations. *J. Pers. Soc. Psychol.* 51 1173–1182. 10.1037/0022-3514.51.6.1173 3806354

[B5] Bechtold J. Cavanagh C. Shulman E. P. Cauffman E. (2013). Does mother know best? Adolescent and mother reports of impulsivity and subsequent delinquency. *J. Youth Adolesc.* 43 1903–1913. 10.1007/s10964-013-0080-9 24337690

[B6] Bollen K. A. (1989). *Structural Equations with Latent Variables.* New York, NY: Wiley.

[B7] Buss A. H. Plomin R. (1984). *Temperament: Early Developing Personality Traits.* Hillsdale, NJ: Erlbaum.

[B8] Campbell D. T. Fiske D. W. (1959). Convergent and discriminant validity by the multitrait-multimethod matrix. *Psychol. Bull.* 56 81–105. 10.1037/h0046016 13634291

[B9] Chassin L. Barrera M. Bech K. Kossak-Fuller J. (1992). Recruiting a community sample of adolescent children of alcoholics: a comparison of three subject sources. *J. Stud. Alcohol* 53 316–319. 10.15288/jsa.1992.53.316 1619925

[B10] Chassin L. Rogosch F. Barrera M. (1991). Substance use and symptomology among adolescent children of alcoholics. *J. Abnorm. Psychol.* 100 449–463. 10.1037/0021-843X.100.4.449 1757658

[B11] Chen H. (1990). *Theory-Driven Evaluations.* Newbury Park, CA: SAGE Publications.

[B12] Cole D. A. Maxwell S. E. (2003). Testing mediational models with longitudinal data: questions and tips in the use of structural equation modeling. *J. Abnorm. Psychol.* 112 558–577. 10.1037/0021-843X.112.4.558 14674869

[B13] De Los Reyes A. Augenstein T. M. Wang M. Thomas S. A. Drabick D. A. G. Burgers D. E. (2015). The validity of the multi-informant approach to assessing child and adolescent mental health. *Psychol. Bull.* 141 858–900. 10.1037/a0038498 25915035 PMC4486608

[B14] De Los Reyes A. Kazdin A. E. (2005). Informant discrepancies in the assessment of childhood psychopathology: a critical review, theoretical framework, and recommendations for further study. *Psychol. Bull.* 131 483–509. 10.1037/0033-2909.131.4.483 16060799

[B15] De Los Reyes A. Thomas S. A. Goodman K. L. Kundey S. M. A. (2013). Principles underlying the use multiple informants’ reports. *Annu. Rev. Clin. Psychol.* 9 123–149. 10.1146/annurev-clinpsy-050212-185617 23140332 PMC4103654

[B16] Dumenci L. (2000). “Multitrait-multimethod analysis,” in *Handbook of Applied Multivariate Statistics and Mathematical Modeling* eds Brown S. D. Tinsley H. E. A. (San Diego, CA: Academic Press) 583–611.

[B17] Eid M. (2000). A multitrait-multimethod model with minimal assumptions. *Psychometrika* 65 241–261. 10.1007/BF02294377

[B18] Eid M. Lischetzke T. Nussbeck F. W. (2006). “Structural equation models for multitrait–multimethod data,” in *Handbook of Multimethod Measurement in Psychology* eds Eid M. Diener E. (Washington, DC: American Psychological Association) 283–299.

[B19] Eid M. Lischetzke T. Nussbeck F. W. Trierweiler L. I. (2003). Separating trait effects from trait-specific method effects in multitrait-multimethod models: a multiple-indicator CT-C(M-1) model. *Psychol. Methods* 8 38–60. 10.1037/1082-989X.8.1.38 12741672

[B20] Eid M. Nussbeck F. W. Geiser C. Cole D. A. Gollwitzer M. Lischetzke T. (2008). Structural equation modeling of multitrait-multimethod data: different models for different types of methods. *Psychol. Methods* 13 230–253. 10.1037/a0013219 18778153

[B21] Fritz M. S. MacKinnon D. P. (2007). Required sample size to detect the mediated effect. *Psychol. Sci.* 18 233–239. 10.1111/j.1467-9280.2007.01882.x 17444920 PMC2843527

[B22] Geiser C. Eid M. Nussbeck F. W. (2008). On the meaning of the latent variables in the CT-C(M – 1) model: a comment on Maydeu-Olivares and Coffman (2006). *Psychol. Methods* 13 49–57. 10.1037/1082-989X.13.1.49 18331153

[B23] Geiser C. Koch T. Eid M. (2014). Data-generating mechanisms versus constructively-defined latent variables in multitrait-multimethod analysis: a comment on Castro-Schilo, Widaman, and Grimm (2013). *Struct. Equ. Modeling* 21 509–523. 10.1080/10705511.2014.919816 25419098 PMC4235740

[B24] Hayes A. F. (2009). Beyond Baron and Kenny: statistical mediation analysis in the new millennium. *Commun. Monogr.* 76 408–420. 10.1080/03637750903310360

[B25] Hayes A. F. Scharkow M. (2013). The relative trustworthiness of inferential tests of the indirect effect in statistical mediation analysis: does method really matter? *Psychol. Sci.* 24 1918–1927. 10.1177/0956797613480187 23955356

[B26] Hightower A. D. Spinell A. Lotyczewski B. S. (1989). *Teacher-Child Rating Scale (T-CRS) Guidelines.* Rochester, NY: Primary Mental Health Project, University of Rochester.

[B27] Hunsley J. Mash E. J. (2007). Evidence-based assessment. *Annu. Rev. Clin. Psychol.* 3 29–51. 10.1146/annurev.clinpsy.3.022806.091419 17716047

[B28] Kazdin A. E. (2007). Mediators and mechanisms of change in psychotherapy research. *Annu. Rev. Clin. Psychol.* 3 1–27. 10.1146/annurev.clinpsy.3.022806.091432 17716046

[B29] Kazdin A. E. (2009). Understanding how and why psychotherapy leads to change. *Psychother. Res.* 19 418–428. 10.1080/10503300802448899 19034715

[B30] Kazdin A. E. (2011). Evidence-based treatment research: advances, limitations, and next steps. *Am. Psychol.* 66 685–698. 10.1037/a0024975 22082384

[B31] Kazdin A. E. Nock M. K. (2003). Delineating mechanisms of change in child and adolescent therapy: methodological issues and research recommendations. *J. Child Psychol. Psychiatry* 44 1116–1129. 10.1111/1469-7610.00195 14626454

[B32] Kraemer H. C. Measelle J. R. Ablow J. C. Essex M. J. Boyce W. T. Kupfer D. J. (2003). A new approach to integrating data from multiple informants in psychiatric assessment and research: mixing and matching contexts and perspectives. *Am. J. Psychiatry* 160 1566–1577. 10.1176/appi.ajp.160.9.1566 12944328

[B33] Little T. D. Cunningham W. A. Shahar G. Widaman K. F. (2002). To parcel or not to parcel: exploring the question, weighing the merits. *Struct. Equ. Modeling* 9 151–173. 10.1207/S15328007SEM0902_1

[B34] MacKinnon D. P. (2008). *Introduction to Statistical Analysis.* New York, NY: Lawrence Erlbaum Associates.

[B35] MacKinnon D. P. Lockhart G. Baraldi A. N. Gelfand L. A. (2013). “Evaluating treatment mediators and moderators,” in *The Oxford Handbook of Research Strategies for Clinical Psychology* eds Comer J. S. Kendall P. C. (New York, NY: Oxford University Press) 262–286.

[B36] MacKinnon D. P. Lockwood C. M. Williams J. (2004). Confidence limits for the indirect effect: distribution of the product and resampling methods. *Multivariate Behav. Res.* 39 99–128. 10.1207/s15327906mbr3901_4 20157642 PMC2821115

[B37] Marsh H. W. Grayson D. (1995). “Latent variable models of multitrait-multimethod data,” in *Structural Equation Modeling: Concepts, Issues, and Applications* ed. Hoyle R. H. (Thousand Oaks, CA: Sage Publications) 177–198.

[B38] Marsh H. W. Hocevar D. (1988). A new, more powerful approach to multitrait-multimethod analyses: application of second-order confirmatory factor analysis. *J. Appl. Psychol.* 73 107–117. 10.1037/0021-9010.73.1.107

[B39] Muthén L. K. Muthén B. O. (1998–2012). *Mplus User’s Guide* 7th Edn Los Angeles, CA: Muthén & Muthén.

[B40] Pohl S. Steyer R. (2010). Modeling common traits and method effects in multitrait-multimethod analysis. *Multivariate Behav. Res.* 45 45–72. 10.1080/00273170903504729 26789084

[B41] Preacher K. J. (2015). Advances in mediation analysis: a survey and synthesis of new developments. *Annu. Rev. Psychol.* 66 825–852. 10.1146/annurev-psych-010814-015258 25148853

[B42] Preacher K. J. Kelley K. (2011). Effect size measures for mediation models: quantitative strategies for communicating indirect effects. *Psychol. Methods* 16 93–115. 10.1037/a0022658 21500915

[B43] Rescorla L. A. Ginzburg S. Achenbach T. M. Ivanova M. Y. Almqvist F. Begovac I. (2012). Cross-informant agreement between parent-reported and adolescent self-reported problems in 25 societies. *J. Clin. Child Adolesc. Psychol.* 42 262–273. 10.1080/15374416.2012.717870 23009025

[B44] Roosa M. W. Wolchik S. A. Sandler I. N. (1997). “Preventing the negative effects of common stressors: current status and future directions,” in *Handbook of Children’s Coping: Linking Theory and Intervention* eds Sandler I. N. Wolchik S. A. (New York, NY: Plenum Press) 515–533.

[B45] Weisz J. R. Gray J. S. (2008). Evidence-based psychotherapy for children and adolescents: data from the present and a model for the future. *Child Adolesc. Ment. Health* 13 54–65. 10.1111/j.1475-3588.2007.00475.x 32847169

[B46] Weisz J. R. Kazdin A. E. (2010). *Evidence-Based Psychotherapies for Children and Adolescents.* New York, NY: Guilford.

[B47] Widaman K. F. (1985). Hierarchically nested covariance structure models for multitrait-multimethod data. *Appl. Psychol. Meas.* 9 1–26. 10.1177/014662168500900101

[B48] Wothke W. (1995). “Covariance components analysis of the multitrait-multimethod matrix,” in *Personality Research, Methods, and Theory: A Festschrift Honoring Donald W. Fiske* ed. Shrout P. E. (Hillsdale, NJ: Lawrence Erlbaum Associates) 125–144.

