# Supplementary material for: Analyzing Statistical Mediation with Multiple Informants: A New Approach with an Application in Clinical Psychology
Source: Front Psychol. 2015 Nov 13;6:1674. doi: 10.3389/fpsyg.2015.01674 (PMC4643137; doi:10.3389/fpsyg.2015.01674)
Supplement: Supplementary file 1 [file Data_Sheet_1.DOCX]

**Appendix A**

Mplus Input File for Specifying the CT-C(M – 1) Mediation Model with Indicator-Specific Traits and a Latent Means Approach

TITLE: Indicator Specific CT-C(M-1) Mediation Model using Latent Means Approach

DATA: file = data.dat;

VARIABLE: names = M3imp1 M3imp2 M3imp3 F3imp1 F3imp2 F3imp3 C3imp1 C3imp2 C3imp3

M3fru1 M3fru2 M3fru3 F3fru1 F3fru2 F3fru3 C3fru1 C3fru2 C3fru3

M3Ext1 M3Ext2 M3Ext3 F3Ext1 F3Ext2 F3Ext3 C3Ext1 C3Ext2 C3Ext3;

ANALYSIS: bootstrap = 1000;

MODEL: ! Impulsivity trait factors

TIMP1 by M3imp1 F3imp1 C3imp1;

TIMP2 by M3imp2 F3imp2 C3imp2;

TIMP3 by M3imp3 F3imp3 C3imp3;

! Frustration tolerance trait factors

TFRU1 by M3fru1 F3fru1 C3fru1;

TFRU2 by M3fru2 F3fru2 C3fru2;

TFRU3 by M3fru3 F3fru3 C3fru3;

! Externalizing trait factors

TEXT1 by M3Ext1 F3Ext1 C3Ext1;

TEXT2 by M3Ext2 F3Ext2 C3Ext2;

TEXT3 by M3Ext3 F3Ext3 C3Ext3;

! Father report method factors

FIMP by F3imp1 F3imp2 F3imp3;

FFRU by F3fru1 F3fru2 F3fru3;

FEXT by F3Ext1 F3Ext2 F3Ext3;

! Child report method factors

CIMP by C3imp1 C3imp2 C3imp3;

CFRU by C3fru1 C3fru2 C3fru3;

CEXT by C3Ext1 C3Ext2 C3Ext3;

! Latent Means (Common) Factors and indicator-specific factors (IS)

IMP by TIMP1@1 TIMP2@1 TIMP3@1;

ISI2 by TIMP1@-1 TIMP2@1;

ISI3 by TIMP1@-1 TIMP3@1;

FRU by TFRU1@1 TFRU2@1 TFRU3@1;

ISF2 by TFRU1@-1 TFRU2@1;

ISF3 by TFRU1@-1 TFRU3@1;

EXT by TEXT1@1 TEXT2@1 TEXT3@1;

ISE2 by TEXT1@-1 TEXT2@1;

ISE3 by TEXT1@-1 TEXT3@1;

! Latent correlations that are zero by definition in the model

TIMP1 TIMP2 TIMP3 with FIMP@0 CIMP@0;

TFRU1 TFRU2 TFRU3 with FFRU@0 CFRU@0;

TEXT1 TEXT2 TEXT3 with FEXT@0 CEXT@0;

IMP ISI2 ISI3 with FIMP@0 CIMP@0;

FRU ISF2 ISF3 with FFRU@0 CFRU@0;

EXT ISE2 ISE3 with FEXT@0 CEXT@0;

! Residual variances that are zero by definition in the model

TIMP1@0; TIMP2@0; TIMP3@0; TFRU1@0; TFRU2@0; TFRU3@0; TEXT1@0; TEXT2@0; TEXT3@0;

! Allowed correlations between method/IS factors and latent residuals

FRU with ISF2* ISF3* ISE2* ISE3* ISI2* ISI3*;

EXT with ISF2* ISF3* ISE2* ISE3* ISI2* ISI3*;

FRU with FIMP FEXT CIMP CEXT;

EXT with FIMP FFRU CIMP CFRU;

! Latent Mediation Model

FRU on IMP; EXT on FRU IMP;

model indirect: EXT ind IMP;

OUTPUT: sampstat stdyx cinterval(bcbootstrap);
